# Risk of Type II Diabetes Mellitus Among B‐Cell Non‐Hodgkin's Lymphoma Survivors

**DOI:** 10.1002/cam4.71367

**Published:** 2026-01-12

**Authors:** Ellenor Chi, Derek K. Chang, Chun‐Pin Esther Chang, Seungmin Kim, Jihye Park, John Snyder, Vikrant Deshmukh, Michael G. Newman, Catherine J. Lee, Mia Hashibe

**Affiliations:** ^1^ Spencer Fox Eccles School of Medicine University of Utah Salt Lake City Utah USA; ^2^ Division of Public Health, Department of Family and Preventive Medicine University of Utah School of Medicine Salt Lake City Utah USA; ^3^ Huntsman Cancer Institute Salt Lake City Utah USA; ^4^ Department of Epidemiology, Gillings School of Global Public Health University of North Carolina at Chapel Hill Chapel Hill North Carolina USA; ^5^ Intermountain Healthcare Salt Lake City Utah USA; ^6^ University of Utah Health Sciences Center Salt Lake City Utah USA; ^7^ Transplant and Cellular Therapy Program Huntsman Cancer Institute, University of Utah School of Medicine Salt Lake City Utah USA; ^8^ Utah Cancer Registry Salt Lake City Utah USA

**Keywords:** B‐cell non‐Hodgkin's lymphoma, cancer survivors, diabetes mellitus, late effects, survivorship

## Abstract

**Purpose:**

Advancing therapies have increased B‐cell Non‐Hodgkin's Lymphoma (B‐NHL) patient survival. However, data are limited on the risk of type II diabetes mellitus (type II DM) in adult survivors following treatment. This study examines the risk of type II DM among a Utah population of B‐NHL survivors, compared to the general population.

**Methods:**

A cohort of 3529 adult survivors diagnosed with B‐NHL in Utah between 1997 and 2013 in the Utah Cancer Registry and 13,339 individuals from the general population were identified using the Utah Population Database (UPDB). Multivariate Cox Proportional Hazard models were used to estimate adjusted hazard ratios (aHR) for developing type II DM, stratified for time post‐diagnosis.

**Results:**

Compared to the cancer‐free population, B‐NHL survivors had an overall increased risk of developing type II DM (HR: 1.49; 95% CI: 1.32, 1.69), largely within the first year (HR: 4.41; 95% CI: 3.52, 5.52) following diagnosis. Older B‐NHL survivors were more likely to develop type II DM at any time compared to survivors < 40 years [40–65 years (HR: 2.66; 95% CI 1.48–4.79); ≥ 65 years (HR: 3.77; 95% CI 2.09–6.78)]. Obese (BMI > 30 kg/m^2^) survivors had a 4.06‐fold increase in the risk of type II DM compared to normal BMI (18–24.9 kg/m^2^) cancer survivors. Cancer treatment did not increase the risk of type II DM compared to no treatment.

**Conclusions:**

Adult B‐NHL cancer survivors were at an overall increased risk of developing type II DM compared to the general population, within the first year and overall, following a cancer diagnosis. This study provides evidence suggesting the importance of obesity prevention and improvement in care management oversight for B‐NHL survivorship and DM outcomes.

## 1 Introduction

B‐cell Non‐Hodgkin's Lymphoma (B‐NHL) is the 7th most common cancer in the United States that includes diffuse large B‐cell lymphoma (DLBCL), follicular lymphoma (FL), mantle cell lymphoma (MCL), and marginal zone lymphoma [1, 2, 3, 4]. In 2022, there were an estimated 80,470 new cases diagnosed (4.2% of all new cancer cases), 20,250 deaths due to B‐NHL (3.3% of all cancer deaths), and approximately 845,550 people living with B‐NHL [5, 6]. The overall 5‐year relative survival rate has been increasing between 1975 and 2017, from 46.64% to 78.14%, mainly attributed to newly developed therapeutics for B‐NHL [7].

Current treatment options for NHL include chemotherapy, radiation, surgery, immunotherapy, and hematopoietic stem cell transplant, which have been shown to increase overall survival (OS) [8]. However, patients who survive treatment may have an increased risk for chronic diseases and cardiovascular events with the above treatment modalities [9, 10, 11, 12]. One meta‐analysis of cross‐sectional studies, which involved a total of 6763 participants (1762 cases and 5001 controls) additionally showed a higher risk of metabolic syndrome among cancer survivors compared to healthy control groups. In a subgroup meta‐analysis by cancer type, an association between hematologic malignancies, including NHL, and metabolic syndrome was observed [13]. To our knowledge, the current literature lacks a large cohort study focused on the risk of type II DM in adult survivors of B‐NHL. Thus, we aimed to analyze the risk for developing type II DM among adult B‐NHL survivors in Utah compared to their age‐sex matched individuals from the Utah general population using data from the Utah Population Database.

## 2 Methods

A population‐based cohort was developed with the Utah Population Database (UPDB) [14, 15, 16], which includes data from the Utah Cancer Registry (UCR), Utah Department of Health (UDOH), electronic health records (EHR), statewide ambulatory surgery and inpatient data, and family medical history. The EHR data was obtained through the University of Utah Health System and Intermountain Healthcare. The utilization of this data was approved by the University of Utah Institutional Review Board and the Resource for Genetic and Epidemiologic Research.

**FIGURE 1:**
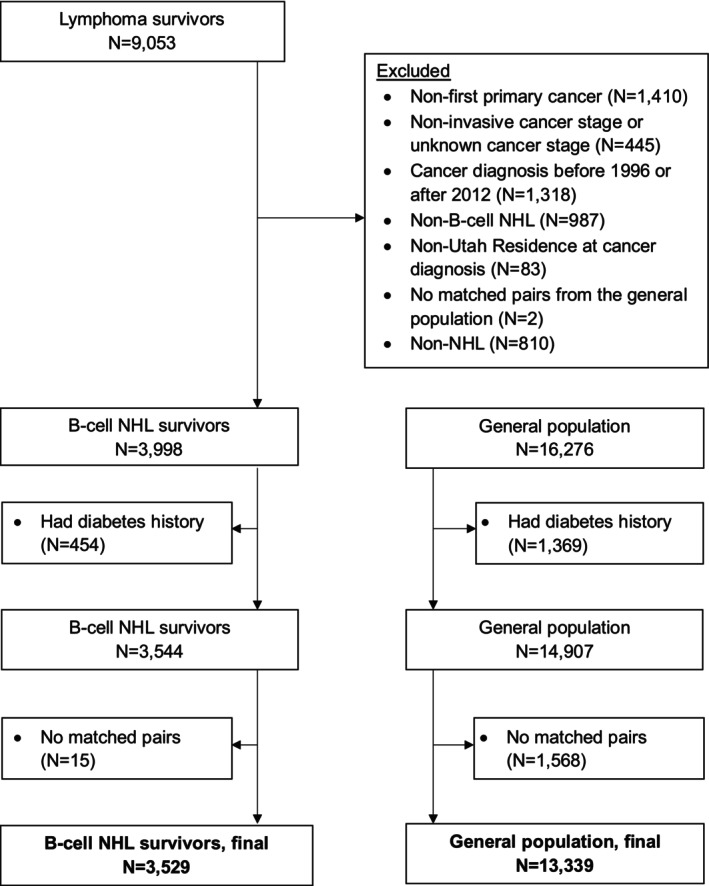
Consort diagram.

The contemporary definition of a cancer survivor is an individual living from the time of a cancer diagnosis until the end of their life [17]. Therefore, our cohort selection included NHL patients aged > 18 at the time of diagnosis (SEER ICD‐O‐3 codes: 33041 and 33042), had a first primary B‐NHL diagnosis between 1997 and 2015 (ICD‐O‐3 morphology codes: 9670, 9671, 9673, 9675, 9679, 9680, 9684, 9687, 9689, 9690, 9691, 9695, 9698, 9699, 9823), lived in the state of Utah at the time of diagnosis, and had a documented cancer stage at diagnosis. The UCR uses the SEER cancer staging system for NHL [18], including the following categories: localized, regional, and distant. We excluded B‐NHL patients for whom there were no eligible individuals to match from the general population (*n* = 2) and B‐NHL patients with in situ or unknown cancer staging (*n* = 445) (Figure 1). Individuals with a diagnosis of DM prior to having B‐NHL diagnosis were also excluded, as well as any individual within the matched cancer‐free population who had a prior DM diagnosis was excluded. Each B‐NHL survivor was matched up to 5 individuals from the general population cohort by birth year, birth state, and sex.

The primary outcome of interest in this study was an incident diagnosis of type II DM following a B‐NHL diagnosis. DM diagnosis was defined by diabetes type (e.g., type I DM, type II DM) using the International Classification of Diseases, Ninth Revision (ICD‐9) codes (ICD‐9 codes: 250.00–250.93). Secondary diabetes mellitus and abnormal findings related to diabetes (ICD‐9 codes: 790.2–790.29, 791.5, 791.6, V45.85, V53.91, and V65.46) were classified as ‘other’ in the analysis. New type II DM diagnoses were identified for all time, 0–1 year, 1–5 years, 5–10 years, and greater than 10 years following a B‐NHL diagnosis. Follow‐up time was calculated between the time from initial cancer diagnosis to the date of type II DM diagnosis. For individuals who were not diagnosed with type II DM, the follow‐up time was calculated between the time of cancer diagnosis of the matched cancer patient to the last date of Utah residence or date of death. Matched cancer‐free persons were followed from the date of diagnosis of their matched NHL case and follow‐up was monitored in the identical way.

Chi‐squared tests were used to compare differences in the distribution of baseline characteristics, including demographics and vital status, between B‐NHL cancer survivors and the individuals from the cancer‐free population. Multivariate Cox proportional hazards models stratified on matched pairs were used to calculate estimate hazard ratios (HRs) and 95% confidence intervals (95% CI) for the risk of Type II DM, across all specified time periods. Multivariate models were adjusted for the matched factors (birth year, birth state, and sex), as well as race, baseline BMI, and Charlson Comorbidity Index (CCI) scores [19]. Diabetes was excluded in the CCI calculation to avoid double counting. In addition, Cox proportional hazard models were used to account for diagnosis year, age at diagnosis, cancer stage at diagnosis, race, BMI at baseline, and baseline CCI score among B‐NHL survivors. We tested the proportionality of hazards in all Cox models by fitting product terms between covariate and time. In cases where proportionality of the hazards did not hold, Cox models with cubic spline functions were used to report the estimates from the cubic spline function.

Baseline BMI values were calculated from self‐reported height and weight from the driver license records. We obtained the first available BMI before the cancer diagnosis date (or the cancer diagnosis date for matched cases of the general population) for analysis. The BMI data ranged from 1968 to 2014 (median: year 2003), covering a period from 1 day before cancer diagnosis to 33.9 years before diagnosis (median: 2.9 years before cancer diagnosis). About 20% of the cohort had missing BMI data. Participants with missing BMI values had BMI scores imputed using the multiple imputation by the fully conditional specification approach [20], which included the following baseline characteristics as covariates: sex, race, age at cancer diagnosis, cancer status, and baseline CCI score. Sensitivity analysis was conducted by excluding individuals with missing BMI. Clinical characteristic assessment included cancer stage at diagnosis, treatment modality, baseline CCI score, and histology subtype. All analyses were performed using SAS (version 9.4).

## 3 Results

A total of 3529 B‐NHL cancer survivors and 13,339 matched individuals from the general population were included in the final cohort (Figure S1). The median follow‐up was 4.7 years among B‐NHL survivors and 7.9 years among the general population (Table S1). The B‐NHL cancer survivor group had a higher proportion of overweight and obese individuals [BMI > 25 kg/m^2^] (*p* = 0.0004), was less ethnically diverse (*p* < 0.0001), and had a significantly higher mortality (*p* < 0.0001) compared to the general population cohort (Table 1). More than half of B‐NHL patients were 60 or more years old (58.8%) with a median age of 63 years (Table 2). Patients with diffuse large B‐cell lymphoma (DLBCL; 44.9%) and follicular lymphoma (FL; 26.5%) diagnosis represented a significant proportion in this cohort, and most survivors did not receive a stem cell transplant.

**TABLE 1:** Characteristics of B‐cell NHL survivors and their matched cancer‐free cohort.

	B‐cell NHL cases		Cancer‐free		*p* ^a^
	*N* = 3529		*N* = 13,339		
	*N*	%	*N*	%	
Sex					
Male	1940	55.0	7331	55.0	0.9882
Female	1589	45.0	6008	45.0	
Race					
White	3439	97.5	12,436	93.2	**< 0.0001**
Other	88	2.5	522	3.9	
Unknown	< 11	—	381	2.9	
Ethnicity					
Hispanic	252	7.1	815	6.1	**< 0.0001**
Non‐hispanic	3252	92.2	9513	71.3	
Unknown	25	0.7	3011	22.6	
Vital status					
Alive	1976	56.0	10,793	80.9	**< 0.0001**
Dead	1553	44.0	2546	19.1	
Body mass index at baseline					
< 18.5 kg/m^2^	49	1.4	159	1.2	**0.0004**
18.5–24.9 kg/m^2^	1302	36.9	5346	40.1	
25–29.9 kg/m^2^	1451	41.1	5432	40.7	
30+ kg/m^2^	727	20.6	2402	18.0	
Charlson comorbidity index at baseline					
0	2084	59.1	9648	72.3	**< 0.0001**
1	731	20.7	2250	16.9	
2+	714	20.2	1441	10.1	
Family history of any cancer^b^					
No	1475	41.8	5639	42.3	0.6091
Yes	2054	58.2	7700	57.7	
Family history of lymphoma^b^					
No	3336	94.5	12,944	97.0	**< 0.0001**
Yes	193	5.5	395	3.0	
Family history of diabetes^b^					
No	1590	45.1	5811	43.6	0.1124
Yes	1939	54.9	7528	56.4	
Diagnosis of diabetes after diagnosis of NHL					
No	3084	87.39	12,053	90.36	**< 0.0001**
Yes	445	12.61	1286	9.64	
Diabetes					
Type I	< 11	—	39	0.29	0.5104
Type II	375	10.63	1108	8.31	**< 0.0001**

*Note:* Numbers less than 11 have been suppressed to protect confidentiality.

^a^ Two‐sided chi‐square test.

^b^ Includes first, second and third degree relatives.

**TABLE 2:** Clinical characteristics of B‐cell NHL survivors.

	*N* = 3529	
	*N*	%
Age at cancer diagnosis		
18–39 years	305	8.6
40–49 years	418	11.8
50–59 years	730	20.7
60–69 years	861	24.4
70–79 years	762	21.6
80–100 years	453	12.8
*Median = 63.0 years*		
Diagnosis year		
1997–2000	673	19.1
2001–2004	805	22.8
2005–2009	1098	31.1
2010–2015	953	27.0
Cancer stage at diagnosis		
Localized	1148	32.5
Regional	629	17.8
Distant	1752	49.7
Cancer treatment		
No treatment or surgery only	1061	30.1
Radiation only or radiation + surgery	241	6.8
Chemotherapy only or chemo + surgery	1544	43.8
Radiation + chemotherapy or radiation + chemotherapy + surgery	574	16.3
Unknown	109	3.1
Stem cell transplant		
No	3251	92.1
Yes	278	7.9
Cancer histology subtype		
Malignant lymphoma, small B lymphocytic, NOS	186	5.3
Malignant lymphoma, lymphoplasmacytic	38	1.1
Mantle cell lymphoma	181	5.1
Mediastinal large B‐cell lymphoma	12	0.3
Malignant lymphoma, large B‐cell, diffuse, NOS	1584	44.9
Malignant lymphoma, large B‐cell, diffuse, immunoblastic, NOS	76	2.2
Malignant lymphoma, mixed sm. and lg. cell, diffuse	28	0.8
Burkitt lymphoma, NOS	63	1.8
Splenic marginal zone B‐cell lymphoma	35	1.0
Follicular lymphoma	936	26.5
Extranodal marginal zone lymphoma of mucosa‐associated lymphoid tissue (MALT lymphoma)	315	8.9
Chronic lymphocytic leukemia/SLL	75	2.1
Cancer site		
Nodal	2432	68.9
Extra nodal	1097	31.1

Among B‐NHL survivors diagnosed with DM, 375 (10.6%) had type II DM (Table 3). The proportion of type II DM in the B‐NHL cancer survivor group was higher than the general population cohort at all time (10.6% vs. 8.3%) and in the first year after diagnosis of B‐NHL (6.0% vs. 1.1%). The all‐time risk of developing type II DM was higher among B‐NHL patients compared to the cancer‐free population (HR: 1.49, 95% CI: 1.32–1.69) and in the first year post‐diagnosis (HR: 4.41; 95% CI: 3.52, 5.52) (Table 4).

**TABLE 3:** Type II DM diagnosis among B‐NHL survivors versus matched general population cohort.

	Type II DM			
	B‐NHL		General	
	Survivors		Population	
	*N*	%	*N*	%
All‐time	375	10.6	1108	8.3
0–1 year	211	6.0	147	1.1
> 1–5 years	92	2.6	459	3.4
> 5–10 years	46	1.3	355	2.7
> 10 years	26	0.7	147	1.1

**TABLE 4:** Hazard ratios for Type II DM in B‐cell NHL survivors vs. matched general population cohort by follow up time.

	Type II DM	
	HR (95% CI)^a^	*p*
All‐time	1.49 (1.32, 1.69)^b^	< 0.0001
0–1 year	4.41 (3.52, 5.52)^b^	< 0.0001
> 1–5 years	1.01 (0.79, 1.29)	0.7975
> 5–10 years	0.75 (0.53, 1.06)	0.5023
> 10 years	1.07 (0.64, 1.77)	0.6516

^a^ HRs adjusted for sex, race, baseline BMI, baseline CCI score.

^b^ Proportional hazard assumption was violated; Cox models with cubic splines were used.

When patients with missing BMI data were excluded from the analysis (Table S2), the all‐time risk of developing type II DM in B‐NHL patients remained elevated compared to the cancer‐free population (HR 1.49; 95% CI: 1.29–1.73) and in the first year post‐diagnosis (HR 4.11, 95% CI: 3.19–5.29).

Lastly, when death was also assessed as a competing risk for B‐NHL survivors, B‐NHL survivors also had an elevated all‐time risk of developing type II DM compared to a matched cancer‐free population (HR 2.61,95% CI: 2.46–2.77) and in the first year post‐diagnosis (HR 4.88,95% CI: 4.04–5.90) (Table S3).

In multivariable analysis, patients older than 65 years of age were more likely to develop type II DM overall (HR: 3.77; 95% CI: 2.09–6.78; reference < 40 years), in the first year (HR: 4.57; 95% CI: 1.86–11.25) and 1–5 years (HR: 5.79; 95% CI: 1.39–24.03) after cancer diagnosis (Table 5). Those aged 40–65 years were more likely to develop type II DM overall (HR: 2.66; 95% CI: 1.48–4.79; reference < 40 years) and in the first year post‐diagnosis (HR: 3.07; 95% CI: 1.24–7.59). In addition, patients who were male had a higher risk to develop type II DM overall (HR: 1.47; 95% CI: 1.19–1.81; reference females). Furthermore, obesity (BMI > 30 kg/m^2^) also contributed to an overall increased risk of developing type II DM (HR: 4.06; 95% CI: 3.03–5.42; reference BMI 18–24.9 kg/m^2^). Cancer stage at diagnosis, cancer treatment, CCI score and cancer site were not associated with diabetes risk among B‐NHL survivors.

**TABLE 5:** Risk factors for type II DM among NHL B‐cell lymphoma survivors in a population‐based cohort in Utah, by years since cancer diagnosis.

	Demographic risk factors										Clinical risk factors								
	0–1 years			> 1–5 years			All‐time						> 1–5 years			All‐time			
	aHR	(95% CI)	*p*	aHR	(95% CI)	*p*	aHR	(95% CI)	*p*		aHR	(95% CI)	*p*	aHR	(95% CI)	*p*	aHR	(95% CI)	*p*
**Age (years) at diagnosis** ^**a**^										**Cancer stage at diagnosis** ^**d**^									
< 40	Reference			Reference^g^			Reference			Localized	Reference			Reference			Reference		
40–65	**3.07**	**(1.24, 7.59)**	**0.0151**	3.56	(0.85, 14.82)	0.0810	**2.66**	**(1.48, 4.79)**	**0.0011**	Regional	1.13	(0.76, 1.70)	0.5460	1.21	(0.66, 2.23)	0.5391	1.16	(0.86, 1.56)	0.3385
65+	**4.57**	**(1.86, 11.25)**	**0.0009**	**5.79**	**(1.39, 24.03)**	**0.0160**	**3.77**	**(2.09, 6.78)**	**< 0.0001**	Distant	1.17	(0.85, 1.60)	0.3430	1.24	(0.78, 1.98)	0.3574	1.11	(0.88, 1.40)	0.3789
**Race**										**Treatment** ^**e**^									
White	Reference			Reference			Reference			No Treatment	Reference			Reference			Reference		
Other	1.4	(0.66, 2.96)	0.3862	1.44	(0.46, 4.55)	0.5346	1.49	(0.86, 2.60)	0.1560	Radiation	0.69	(0.34, 1.40)	0.3046	0.32	(0.08, 1.37)	0.1238	0.65	(0.39, 1.10)	0.1063
**Sex**										Chemotherapy	1.13	(0.81, 1.56)	0.4820	1.35	(0.81, 2.27)	0.2474	1.19	(0.92, 1.52)	0.1853
Female	Reference			Reference			Reference			Radiation + Chemotherapy	0.91	(0.58, 1.42)	0.6680	0.83	(0.41, 1.67)	0.5917	0.92	(0.66, 1.28)	0.6220
Male	**1.41**	**(1.07, 1.87)**	**0.0151**	**1.99**	**(1.27, 3.10)**	**0.0024**	**1.47**	**(1.19, 1.81)**	**0.0003**	**Stem cell transplant** ^**e**^									
**BMI (kg/m** ^**2**^ **) at baseline** ^**b**^										No	Reference			Reference			Reference		
< 18	1.55	(0.37, 6.47)	0.5510	1.96	(0.26, 14.9)	0.5142	1.63	(0.59, 4.50)	0.3422	Yes	0.66	(0.35, 1.23)	0.1864	1.06	(0.52, 2.18)	0.8674	0.9	(0.60, 1.34)	0.6004
18–24.9	Reference			Reference			Reference			**CCI score at baseline** ^**f**^									
25–29.9	**2.05**	**(1.39, 3.04)**	**0.0003**	1.65	(0.92, 2.97)	0.0939	**1.95**	**(1.46, 2.60)**	**< 0.0001**	0	Reference			Reference			Reference		
30+	**4.12**	**(2.77, 6.11)**	**< 0.0001**	**4.26**	**(2.40, 7.56)**	**< 0.0001**	**4.06**	**(3.03, 5.42)**	**< 0.0001**	1	0.81	(0.56, 1.17)	0.2566	1.51	(0.92, 2.47)	0.1053	0.97	(0.75, 1.26)	0.8153
**Family history of diabetes** ^**c**^										2+	1	(0.71, 1.41)	0.9996	1.29	(0.74, 2.24)	0.3693	1.01	(0.77, 1.33)	0.9332
No	Reference			Reference			Reference			**Cancer site** ^**d**^									
Yes	1.06	(0.80, 1.39)	0.7035	1.49	(0.97, 2.29)	0.0699	1.19	(0.97, 1.47)	0.0950	Nodal	Reference			Reference			Reference		
										Extra nodal	0.97	(0.73, 1.30)	0.8500	0.96	(0.61, 1.49)	0.8381	0.92	(0.74, 1.15)	0.4736

*Note: p*‐values are bold‐faced.

^a^ Adjusted for diagnosis year, race, sex, BMI at baseline, and CCI score.

^b^ Adjusted for diagnosis year, age at diagnosis, race, sex, and CCI score.

^c^ Adjusted for sex and race.

^d^ Adjusted for diagnosis year, age at diagnosis, race, sex, BMI at baseline, and CCI score.

^e^ Adjusted for diagnosis year, age at diagnosis, race, sex, BMI at baseline, cancer stage at diagnosis, and CCI score.

^f^ Adjusted for diagnosis year, age at diagnosis, race, sex, and BMI at baseline.

^g^ Proportional hazard assumption was violated; Cox models with cubic splines were used.

## 4 Discussion

In this large population‐based cohort of B‐NHL survivors, we observed a 4.41‐fold increased risk of developing type II DM within the first year post‐diagnosis. These results contribute important information to the existing literature regarding the risk of developing type II DM in adult B‐NHL survivors. The majority of previous studies had focused on patients with a prior diagnosis of DM before NHL, childhood cancer survivors and their DM risks, and the development of metabolic syndromes grouped in a small cohort of multiple hematologic malignancies, including NHL [13, 20, 21, 22, 23, 24, 25]. The lack of adult B‐NHL focused studies on DM risk could be attributed to the difficulty of tracking trends for a hematological cancer type with complex physiology and mechanisms promoting uncontrolled proliferation of clonal B‐cells [26], as well as limited access to a significantly large population size to evaluate this cancer population.

This is the first large cohort study to examine the risk of type II DM onset between adult B‐NHL survivors and matched individuals in the cancer‐free population. However, it is important to note the overall adjusted hazard ratio in the additional follow‐up time periods (i.e., 1–5, 5–10, > 10 years) was not significant likely due to the low number of events captured in the longer follow‐up periods. The first year after cancer diagnosis is a critical treatment period where patients experience toxicities related to lymphoma treatment. However, our results did not show that patients who received chemotherapy or radiation treatment had an increased risk of developing DM compared to patients who received no treatment. These findings align with other studies focusing on risk factors for metabolic syndrome [13]. Other studies have reported an increased risk between treatment with R‐CHOP (rituximab, cyclophosphamide, hydroxydaunorubicin, oncovin, and prednisone), abdominal radiation, or HSCT and the development of insulin insufficiency and resistance [9, 11, 27, 28, 29]. The conflict could be attributed to the complex, heterogeneous mechanisms of B‐NHL, where studying specific trends in secondary diseases is multifactorial. These factors may include accumulation of multivariate cytogenetic aberrations [26], existing comorbidities (e.g., obesity, heart disease, autoimmune diseases), environmental effects, and nutritional/physical lifestyle patterns [6]. It has also been theorized that prolonged hyperinsulinemia, hyperglycemia, cytokine over‐secretion and upregulation of IGF‐1 favors malignant transformation of cells [21, 25]. Notably, these mechanisms are proposed for patients with pre‐existing DM who later develop NHL or other hematologic malignancies [21, 22, 25, 30]. However, a similar biological profile may be present in NHL survivors exposed to treatment and could conversely mediate development of DM. Immune dysregulation, which often drives development of NHL could occur with cytokine over‐secretion and may drive metabolic dysfunction. A recent study investigating differentially expressed genes in patients with type II DM compared to those with NHL showed dysregulation of certain immune‐related pathways in both groups [31].

While not labelled as chemotherapy or radiation treatment, some patients may have been exposed to high‐dose glucocorticoids as part of their lymphoma treatment plan. Though difficult to predict in specific patients, glucocorticoid‐induced DM is a known complication and may mediate the development of DM in patients with lymphoid malignancies, including NHL [11, 32, 33, 34, 35].

The results also show that more B‐NHL patients who developed type II DM after diagnosis were overweight (BMI 25 to 29.9) and obese (BMI > 30 kg/m^2^) at baseline. Although obesity is a well‐known independent risk factor for developing DM [36, 37], this finding also suggests that having a BMI higher than normal range is a risk factor for DM among B‐NHL survivors and lends support for appropriate diet and exercise modifications during active treatment and in post‐treatment follow‐up.

In addition, older age at diagnosis appears highly associated with DM. Most patients diagnosed with B‐NHL were > 65 years old. Because the risk of developing B‐NHL also increases with age, it is of particular interest to evaluate this population's risk of developing DM after cancer diagnosis. Patients > 65 years have limited treatment options due to a greater risk for therapeutic toxicities, including cardiotoxicity and glucocorticoid‐induced DM [11, 38]. Although there have been conflicting results regarding post‐treatment induced DM [9, 11, 25, 30, 31, 32], the increasing fragility of elderly patient physiology may act as a key compounding DM risk factor, which includes decreased glucose metabolism, elevated blood pressure, and increased vascular stiffening [6].

There are inherent limitations with this study design. A major limitation of this study was our inability to extract data related to specific drugs and doses used for treatment of B‐NHL. Understanding the association between certain therapeutic agents and risk for type II DM is important to help counsel patients and appropriately monitor for the long‐term toxicities/late effects of treatment exposures. However, our analysis did not show any overall increased risk for type II DM among patients who received radiation, chemotherapy or both versus no treatment for B‐NHL. Furthermore, we were not able to identify patients who received glucocorticoids as part of their treatment plan, an exposure commonly correlated with DM. Future studies that evaluate these risk factors may be able to better delineate the risk of type II DM development in NHL survivors in groups based on chemotherapy or medication exposures.

Additionally, the use of the ICD‐9 codes between different electronic health record (EHR) systems, utilized by the two largest health care providers in the state of Utah, may contribute to variations in reported DM diagnoses. Although there is a growing global consensus to use interoperable language for medical classifications within EHRs, there are issues where competing EHR systems and different health care organizational structures foster incongruent reporting and inaccurate medical coding [39]. However, the errors encountered in ICD‐9 reporting would not be expected to be different between B‐NHL survivors and the general population. Next, it is important to consider whether DM diagnoses were accurately captured and reported completely. As referenced by the Centers for Disease Control and Prevention (CDC), a large proportion of the population, about 84.1 million Americans, have undiagnosed prediabetes [40]. Prediabetes is unlikely to be coded consistently as there are few ICD‐9 codes available to capture pre‐diabetes as a diagnosis. Despite these limitations using the UPDB as the centralized database, data are consistently being updated and can be re‐analyzed on a regular basis.

There may be surveillance bias. Cancer patients are typically followed more closely, compared to the healthy general population. Since cancer patients require frequent follow‐up visits and more diagnostic tests, secondary diseases may be identified sooner than in a healthy individual. This may be the mechanism behind an observed increase in risks of DM for specific groups of B‐NHL survivors in our analysis.

Lastly, this study may not be generalizable to many patient populations. All patient data is drawn from Utah databases, which have a generally homogenous population skewed towards a majority white population.

This study also has a number of strengths that elucidate the associations between B‐NHL and DM. The large sample size (> 3,500 B‐cell cancer survivors and > 13,000 individuals from the general population), and the use of a centralized statewide EHR database provide extensive longitudinal power to study the effects of cancer in the population. In addition, the large amount of EHR data, which were compiled from the two largest healthcare systems in the state of Utah—including extensive longitudinal ambulatory surgery and inpatient data—allowed a robust analysis of the long‐term health effects of many diseases, disorders, and cancers. Further, the data set used in this study does not contain self‐reported data, in contrast to numerous pieces of cancer survivor literature utilizing self‐reported information [41]. Using a centralized EHR database serves to minimize survival error, reduce recall bias, and strengthen this study's internal validity.

Although using a large population cohort from the UPDB provides strong statistical power, further longitudinal research is necessary to identify trends in different ethnic and racial groups to ensure generalizability. This study showed that B‐NHL survivors, compared to the cancer‐free population, were at a higher risk of developing DM overall after diagnosis. These findings contribute new survivorship data that is otherwise lacking in the current literature. It is important to improve survivorship guidelines for adult B‐NHL patients, who are at an increased risk of developing chronic diseases, including type II DM.

## Author Contributions

**Ellenor Chi:** conceptualization (equal), writing – original draft (equal), writing – review and editing (equal). **Derek K. Chang:** conceptualization (equal), writing – original draft (equal), writing – review and editing (equal). **Chun‐Pin Esther Chang:** conceptualization (equal), formal analysis (equal), investigation (equal), writing – review and editing (equal). **Seungmin Kim:** formal analysis (equal), investigation (equal). **Jihye Park:** formal analysis (equal), investigation (equal). **John Snyder:** data curation (equal). **Vikrant Deshmukh:** data curation (equal). **Michael G. Newman:** data curation (equal). **Catherine J. Lee:** conceptualization (equal), investigation (equal), methodology (equal), supervision (equal), writing – original draft (equal), writing – review and editing (equal). **Mia Hashibe:** conceptualization (equal), formal analysis (equal), investigation (equal), methodology (equal), writing – original draft (equal), writing – review and editing (equal).

## Funding

This work was supported by grants from the NIH (R21 CA185811, R03 CA159357, M. Hashibe, PI), the Huntsman Cancer Institute, Cancer Control and Population Sciences Program, with additional support from the Utah State Department of Health and the University of Utah. The Utah Cancer Registry is funded by the National Cancer Institute‘s SEER Program, Contract No. HHSN261201800016I, the US Center for Disease Control and Prevention‘s National Program of Cancer Registries, Cooperative Agreement No. NU58DP007131, with additional support from the University of Utah and Huntsman Cancer Foundation and also the partial support for the UPDB through grant P30 CA2014 from the National Cancer Institute, University of Utah and from the University of Utah‘s program in Personalized Health and Center for Clinical and Translational Science.

## Ethics Statement

The Institutional Review Board at the University of Utah approved this study (IRB# 65816) and granted a waiver of informed consent.

## Conflicts of Interest

The authors declare no conflicts of interest.

## Supporting information


**Data S1:** cam471367‐sup‐0001‐DataS1.docx.

## Data Availability

Data used for this study can be accessed through the approval of the Resource for Genetic and Epidemiologic Research Committee (RGE), the oversight committee for the UPDB and IRB.
