# Supplementary material for: Risk of Type II Diabetes Mellitus Among B‐Cell Non‐Hodgkin's Lymphoma Survivors
Source: Cancer Med. 2026 Jan 12;15(1):e71367. doi: 10.1002/cam4.71367 (PMC12793787; doi:10.1002/cam4.71367)
Supplement: Supplementary file 1 — Data S1: cam471367‐sup‐0001‐DataS1.docx. [file CAM4-15-e71367-s001.docx]

**General population, final**

**N=13,339**

**B-cell NHL survivors, final**

**N=3,529**

- No matched pairs (N=1,568)
- No matched pairs (N=15)

General population

N=14,907

B-cell NHL survivors

N=3,544

- Had diabetes history (N=1,369)
- Had diabetes history (N=454)

General population

N=16,276

B-cell NHL survivors

N=3,998

Lymphoma survivors

N=9,053

Excluded

- Non-first primary cancer (N=1,410)
- Non-invasive cancer stage or unknown cancer stage (N=445)
- Cancer diagnosis before 1996 or after 2012 (N=1,318)
- Non-B cell NHL (N=987)
- Non-Utah Residence at cancer diagnosis (N=83)
- No matched pairs from the general population (N=2)
- Non-NHL (N=810)

**Figure 1. Consort diagram**

**Supplemental Table 1. Sample size and follow-up time**

|  | **Sample size** | | | **Follow-up year for B-NHL survivors** | | | | | **Follow-up year for general population** | | | | |
| --- | --- | --- | --- | --- | --- | --- | --- | --- | --- | --- | --- | --- | --- |
|  | **B-NHL Survivors** | **General Population** | **Overall** | **Min.** | **Max.** | **Median** | **Mean** | **SD** | **Min.** | **Max.** | **Median** | **Mean** | **SD** |
| **All-time** | 3,529 | 13,339 | 16,868 | 0.0 | 20.2 | 4.7 | 5.8 | 5.1 | 0.0 | 20.2 | 7.9 | 8.5 | 4.9 |
| **0-1 year** | 3,529 | 13,339 | 16,868 | 0.0 | 1.0 | 1.0 | 0.8 | 0.3 | 0.0 | 1.0 | 1.0 | 1.0 | 0.1 |
| **>1-5 years** | 2,681 | 13,095 | 15,776 | 1.0 | 5.0 | 5.0 | 4.3 | 1.2 | 1.0 | 5.0 | 5.0 | 4.5 | 1.0 |
| **>5-10 years** | 1,696 | 9,459 | 11,155 | 5.0 | 10.0 | 9.5 | 8.6 | 1.6 | 5.0 | 10.0 | 10.0 | 8.7 | 1.6 |
| **>10 years** | 759 | 4,749 | 5,508 | 10.0 | 20.2 | 13.2 | 13.7 | 2.7 | 10.0 | 20.2 | 13.7 | 14.1 | 2.8 |

**Supplemental Table 2. Hazard Ratios for DM in B-cell NHL survivors vs. matched general population cohort, excluding individuals with missing BMI**

|  | **Diabetes mellitus (DM)** | **Type II DM** |
| --- | --- | --- |
|  | **HR (95%CI)** | **HR (95%CI)** |
| **All-time** | **1.61 (1.41, 1.84)^a^** | **1.49 (1.29, 1.73)^a^** |
| **0-1 year** | **4.45 (3.51, 5.65)^a^** | **4.11 (3.19, 5.29)^a^** |
| **>1-5 years** | 1.13 (0.86, 1.48) | 0.96 (0.71, 1.30) |
| **>5-10 years** | 0.86 (0.57, 1.29) | 0.65 (0.40, 1.05) |
| **>10 years** | 1.29 (0.50, 3.38) | 1.41 (0.52, 3.80) |
| * HRs adjusted for sex, race, baseline BMI, baseline CCI score  ^a^ Proportional hazard assumption was violated; Cox models with cubic splines were used. | | |

**Supplemental Table 3. Hazard Ratios for DM in B-cell NHL survivors vs. matched general population cohort, death as the competing risk**

|  | **Diabetes mellitus (DM)** | **Type II DM** |
| --- | --- | --- |
|  | **HR (95%CI)** | **HR (95%CI)** |
| **All-time** | **2.59 (2.44, 2.74)^a^** | **2.61 (2.46, 2.77)^a^** |
| **0-1 year** | **5.16 (4.30, 6.20)** | **4.88 (4.04, 5.90)** |
| **>1-5 years** | 1.03 (0.85, 1.25) | 0.93 (0.76, 1.15) |
| **>5-10 years** | 0.80 (0.61, 1.05) | 0.72 (0.53, 0.97) |
| **>10 years** | 0.94 (0.63, 1.38) | 1.00 (0.67, 1.51) |
| * HRs adjusted for sex, race, baseline BMI, baseline CCI score  ^a^ Proportional hazard assumption was violated; Cox models with cubic splines were used. | | |
